# Supplementary material for: Simplification of a registry-based algorithm for ejection fraction prediction in heart failure patients: Applicability in cardiology centres of the Netherlands
Source: PLoS One. 2024 Nov 5;19(11):e0310023. doi: 10.1371/journal.pone.0310023 (PMC11537407; doi:10.1371/journal.pone.0310023)
Supplement: S1 Table — (DOCX) [file pone.0310023.s001.docx]

**S1 Table. Proportion of missing values (%) in each variable of the algorithm among the included patients.**

| **Variable** | **% of missing values** |
| --- | --- |
| **Age** | 0.00 |
| **Sex** | 0.00 |
| **Heart rate** | 16.17 |
| **Systolic blood pressure** | 8.02 |
| **Diastolic blood pressure** | 7.93 |
| **Body mass index** | 7.87 |
| **eGFR** | 37.61 |
| **Ischemic heart disease** | 0.00 |
| **Anemia** | 39.17 |
| **Atrial fibrillation** | 0.00 |
| **Diabetes** | 1.54 |
| **Hypertension** | 1.42 |
| **Valvular disease** | 0.00 |
| **RAAS agents** | 2.65 |
| **Beta-blockers** | 2.65 |
| **Diuretics** | 2.65 |
| **MRA** | 2.65 |
| **Digoxin** | 2.65 |

List of abbreviation: eGFR: estimated glomerular filtration rate. RAAS agents: renin angiotensin aldosterone system.
