# Supplementary material for: Simplification of a registry-based algorithm for ejection fraction prediction in heart failure patients: Applicability in cardiology centres of the Netherlands
Source: PLoS One. 2024 Nov 5;19(11):e0310023. doi: 10.1371/journal.pone.0310023 (PMC11537407; doi:10.1371/journal.pone.0310023)
Supplement: S2 Table — (DOCX) [file pone.0310023.s002.docx]

**S2 Table – Internal validation of the models**

| **Internal validation** | **C-statistic** |
| --- | --- |
| **EF ≥50%** | 0.72 (0.71 – 0.73) |
| **EF ≥40%** | 0.69 (0.68 – 0.70) |
| **EF <50%** | 0.72 (0.71 – 0.73) |
| **EF<40%** | 0.69 (0.68 – 0.70) |
| **Multinomial model** |  |
| **HFmrEF vs HFrEF** | 0.85 (0.84 – 0.86) |
| **HFmrEF vs HFpEF** | 0.19 (0.18 – 0.20) |
| **HFpEF vs HFrEF** | 0.70 (0.70 – 0.71) |
