# Supplementary figures and images for: Simplification of a registry-based algorithm for ejection fraction prediction in heart failure patients: Applicability in cardiology centres of the Netherlands
Source: PLoS One. 2024 Nov 5;19(11):e0310023. doi: 10.1371/journal.pone.0310023 (PMC11537407; doi:10.1371/journal.pone.0310023)

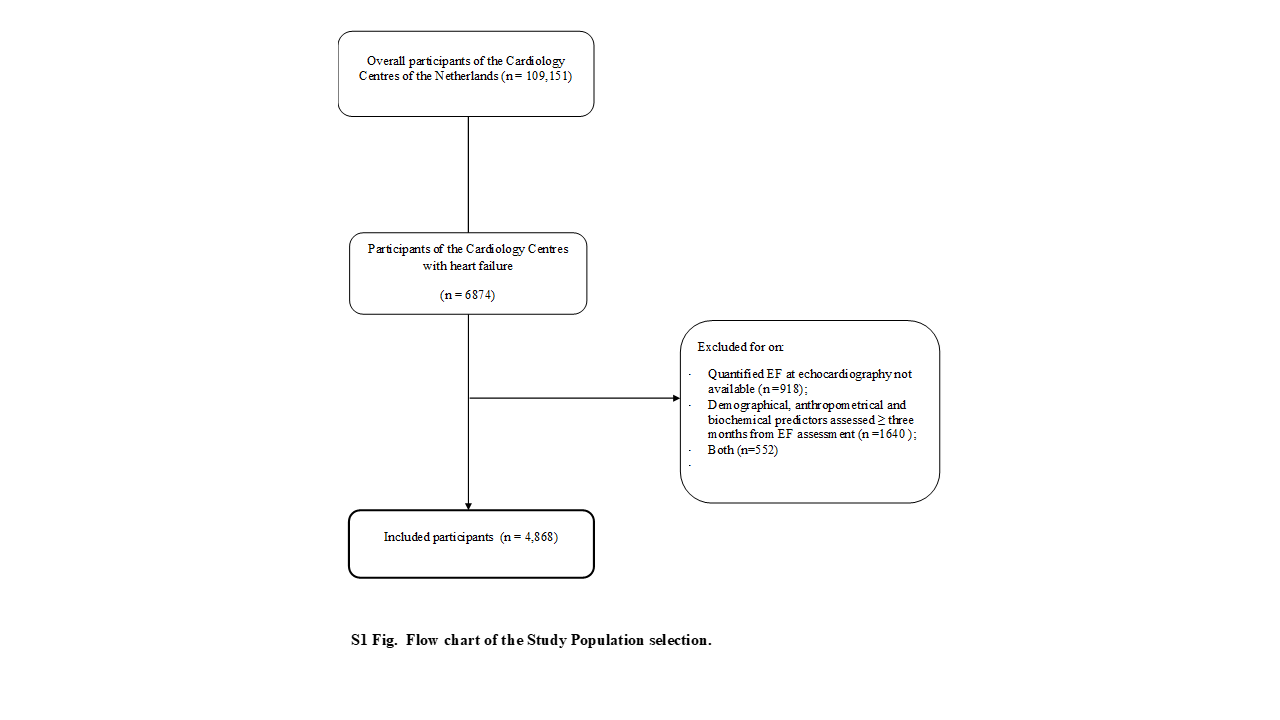

Supplement: S1 Fig — (TIF) [file pone.0310023.s003.tif]
