# Supplementary material for: Simplification of a registry-based algorithm for ejection fraction prediction in heart failure patients: Applicability in cardiology centres of the Netherlands
Source: PLoS One. 2024 Nov 5;19(11):e0310023. doi: 10.1371/journal.pone.0310023 (PMC11537407; doi:10.1371/journal.pone.0310023)
Supplement: Attachment — Submitted filename: Rebuttal letter.docx [file pone.0310023.s005.docx]

**Submission ID**: PONE-D-24-04912R1

**Manuscript title**: Simplification of a Registry-Based Algorithm for Ejection Fraction Prediction in Heart Failure Patients: Applicability in Cardiology Centers of the Netherlands

**Response to editor and reviewers**

We would like to thank the reviewers and editor very much for carefully reviewing our manuscript. We appreciate their detailed review and the opportunity to improve our manuscript. We have addressed all comments below, point-by-point. In addition, we have revised the manuscript (changes are marked in blue text).

**Reviewer #1**: I thank the authors for replying and integrating my comments.

As it is I have no further comments for the authors.

Reply: We are pleased to know that our rebuttal addressed the reviewer’s concerns satisfactorily. Thank you for your thorough review and valuable feedback on our manuscript.

**Reviewer #2**: I thank the authors for addressing the previous comments. I have the following comments remaining:

1. The numbers in Table 3 seem inconsistent. For example, since LVEF >=50% has 0.70 sensitivity, shouldn't I expect LVEF <50% to have 0.7 specificity? (since TP and FN for LVEF>=50% should be the same as TN and FP for LVEF<50%). Rather, it seems the LVEF<50% column contains the FNR (1-specificity) instead of sensitivity and the FPR (1-sensitivity) instead of specificity. There are similar apparent inconsistencies for PPV and NPV in Table 3 and also for the C-statistics in Supplementary Table S2.

**Reply**: Thank you for your valuable feedback. We have carefully reviewed your comment regarding the inconsistencies in Table 3 and the C-statistics in Supplementary Table S2. We acknowledge the importance of accurately presenting these metrics, and apologize for the errors in reporting these results. Upon re-evaluation of our calculations and the analysis of both LVEF >= 50% and LVEF < 50%, as well as LVEF >= 40% and LVEF < 40%, we corrected Table 3 and Supplementary Table S2. The corrected values now ensure the accuracy of our diagnostic performance metrics.

2. The omission of ARNi from the model strikes me as strange. Of the drugs that could be included in the RASi-variable (ACEi, ARB, and ARNi), ARNi is likely the far best at distinguishing between the EF phenotypes. Moreover, ARNi technically includes an ARB. How much this omission affects model performance is difficult to speculate without knowing the time period of enrollment and how many patients used ARNi. I highly suggest to consider including it in the RASi variable, as well as to disclose ACEi/ARB and ARNi separately in the characteristics table.

Reply: Thank you for your insightful feedback regarding the inclusion of ARNi in the RAAS agents variable. Our decision of not including them was based on the fact that the original algorithm developed in the Swedish Heart Failure Registry did not include ARNi due to the timeframe of the study and the adoption of these medications during that period. Specifically, the first prescriptions of ARNi in Sweden began in April 2016, and the study from A. Uijl included patients between 2000 and 2012.

In light of your comment, we have additionally checked our dataset from the Cardiology Centers of the Netherlands about the use of ARNi, but we did not find any mention of it. Upon consultation with the cardiologists at CCN, it was confirmed that regular prescription of ARNi, began only around 2021, while our analysis includes participants from 2007 to 2018.

Therefore, because our study period predates the widespread use of ARNi, incorporating them in the RAAS agents variable is unfortunately not feasible due to the lack of data.

Finally, we would like to point out that ARNi is now being recognized for its benefits across all HF phenotypes, including HFpEF and HFmrEF, as demonstrated in the 2023 PARAGLIDE trial. We understand the potential impact of ARNi and will consider this in future analyses, as more recent data becomes available.

3. Please clarify in the methods between which dates these patients had their index dates. It says CCN data were accessed between Feb 2022 and Jan 2024, but I interpret this literally as data access and not when the patients presented in the clinic.

Reply: we agree with the reviewer that this is an important information and should be clearly stated in the manuscript. Our analysis includes patients who were diagnosed with HF between June 2007 and February 2018. We have added this information in the methods section on page 4.

4. Page 11: "While we acknowledge that the dosage of these medications may be subject to adjustments over time as part of HF management, it is unlikely that the discontinuation or initiation of these drugs would occur significantly long after the initial diagnosis of HF. This is because these HF medications represent the cornerstone of HF treatment and are typically prescribed and monitored closely in clinical practice." This sentence seems at odds with observations from several health care systems. E.g. in EVOLUTION-HF (10.1016/j.jchf.2022.08.009) 33-38% of patients discontinued ACEi/ARB within 12 months.

Reply: We appreciate your observation regarding the discontinuation rates of ACEi/ARB in HF patients, as highlighted in the EVOLUTION-HF study. We recognize that our statement may not align with these observations. Accordingly, we have revised this paragraph as follows, and included a reference to the EVOLUTION-HF study (page 11).

“We acknowledge that the dosage of these medications may be subject to adjustments over time as part of HF management, and discontinuation and initiation of these drugs might as well occur at notable rates. For instance, the EVOLUTION-HF study reported that 33-38% of patients discontinued ACE-inhibitors and angiotensin receptor blockers within 12 months [16]. However, these medications still represent the cornerstone of HF treatment and are typically prescribed and monitored closely in clinical practice, especially in the initial stages following diagnosis.”

5. I suggest to change the term "mid-range" EF to "mildly reduced" EF to align with currently accepted terminology.

Reply: we have revised this accordingly.

6. Conclusion: Just a minor comment but was the distinction between primary care and GP settings made on purpose? "[...] where direct measurements of EF are not routinely available, such as in primary care, or other non-cardiology health settings such as GP settings."

Reply: We acknowledge that the terms "primary care" and "GP settings" can be used interchangeably, and the distinction we made was not intentional. Both terms refer to the settings where general practitioners provide initial and ongoing patient care. We have revised the conclusion to use consistent terminology to avoid confusion: "…where direct measurements of EF are not routinely available, such as in primary care settings."
